# Bullous herpes zoster masquerading as a bullous pemphigoid flare: A case report

**DOI:** 10.1016/j.idcr.2023.e01846

**Published:** 2023-07-07

**Authors:** Ahmed Shehadah, Alshaima Yousef, Anas Hashem, Amir A. Mahmoud, Abhimanyu Aggarwal

**Affiliations:** a Department of Medicine, Rochester General Hospital, NY, USA; b Department of Infectious Diseases, Rochester General Hospital, NY, USA

**Keywords:** Zoster, Disseminated zoster, Bullous herpes, Disseminated herpes zoster, Varicella Zoster Virus, Bullous pemphigoid, Atypical, Pemphigus vulgaris, Shingles

## Abstract

Herpes zoster (HZ) results from the reactivation of dormant varicella zoster virus (VZV) in the posterior dorsal root ganglia manifesting as painful vesicles along the dermatomal distribution as shingles. The risk of reactivation is higher in immunocompromised patients. Herpes zoster lesions can have varied presentations including bullous forms, may induce BP flare, may co-exist in the same lesions, and should be included in the list of differentials for BP flares not responding to the standard of care. Here, we describe a case of a 74-year-old female with a history of recurrent bullous pemphigoid (BP) flares, who was on mycophenolate mofetil, and presented with skin lesions on her right thigh which were typical for her BP flares. Unlike prior flares, the lesions did not respond to prednisone therapy. Her hospitalization course was complicated by encephalopathy. Intravenous acyclovir was started empirically. Viral cultures and polymerase chain reaction from the lesions came back positive for VZV, but the patient succumbed to her illness shortly afterward.

## Introduction

Varicella zoster virus (VZV) is a DNA virus acquired commonly during childhood and can stay dormant in the dorsal root ganglion until reactivation takes place. Herpes zoster (HZ) results from the reactivation of VZV predominantly in immunocompromised individuals. Reactivation manifests commonly as shingles lesions along the dermatomal distribution as painful clustered vesicles on an erythematous base. It rarely presents as bullae or evolves rapidly into disseminated herpes zoster (DHZ) [1]. It is important for clinicians to be aware that shingles may have atypical appearing lesions and HZ should be a part of our differential diagnosis especially in immunocompromised patients or patients with other concurrent skin conditions. Timely diagnosis and treatment of HZ are associated with a reduction in mortality [2]. Here, we have described an atypical case of bullous herpes zoster that was masquerading as a bullous pemphigoid (BP) flare.

## Case report

A 74-year-old female presented to the emergency department with widespread pruritic, painful skin lesions, dyspnea, confusion, and a syncopal episode. Past medical history included BP with multiple BP flares, chronic kidney disease (CKD) stage IV, hypertension, and type 2 diabetes mellitus.

The patient takes several chronic medications. These include acetaminophen 500 mg as needed; amlodipine 5 mg daily; calcitriol 0.25 mcg twice a week; hydroxyzine 10 mg every 8 h as needed for anxiety and itchiness; insulin glargine 25 units at bedtime; melatonin 3 mg at bedtime as needed; and mycophenolate 500 mg twice daily.

In addition, the patient visited her dermatologist five days prior to presenting to the ED and was prescribed several new medications. These included prednisone 20 mg daily, to be gradually tapered; clobetasol topical ointment 0.05% twice daily on her right thigh; and miconazole topical cream 2% twice daily on her right thigh.

Her usual BP lesions included tense and flaccid bullae ranging from 5 to 10 mm in size, mostly populated over her abdomen, anterior thighs, and posterior upper arms. Her BP flares usually affected her right upper thigh, groin, and lower back and manifested as multiple vesicular and vesiculopustular lesions with an erythematous base. She had a BP flare 2 weeks prior to this presentation and was prescribed a prolonged oral prednisone taper (40 mg daily for 4 weeks, and then decreased dose by 10 mg every fourth week) by her dermatologist. But unlike her previous flares, the lesions did not respond to prednisone therapy, and she presented on current admission with several erosive vesicles on an erythematous base, predominantly concentrated over the sacral region and right thigh and some also scattered over her abdomen and face.

Vital signs included blood pressure 125/64 mmHg, pulse 102 bpm, respiratory rate 16/minute saturating 98% on room air, and temperature 97.2°F. Pertinent exam findings included sparse scattered non-erythematous erosive lesions on her face, white densely populated similar appearing lesions over her lower back, right lower abdomen, and right thigh circumferentially. Some of these lesions were escharesque and crusted, while others were oozing yellow exudate. Her mentation and other organ systems were unremarkable on examination.

Pertinent labs included white cell count 11,900/microL with 87% neutrophils, hemoglobin 11.2 g/dL, sodium 135 mEq/L, potassium 4.1 mEq/L, bicarbonate 15 mEq/L, creatinine 3.2 mg/dL (which was her baseline). Two sets of blood cultures stayed negative. Superficial skin cultures from the thigh wound grew *Corynebacterium species* and *Streptococcus dysgalactiae*. She was given a one-time intravenous vancomycin (1000 mg) initially which was adjusted with fluctuating renal function and started on piperacillin-tazobactam (2250 mg every 6 h). Prednisone was decreased to 20 mg daily.

On day 2 of hospitalization, she exhibited progressive fatigue, intermittent confusion and new-onset auditory hallucinations. CT head with contrast did not show any anomalies. MRI brain could not be done due to behavioral limitation with delirium. She was then evaluated by in-house dermatologist who recommended empiric initiation of oral valacyclovir (500 mg twice daily) with a suspicion for herpes zoster. On day 4, the mentation and renal function worsened. She had to be intubated for airway protection, and temporary dialysis was initiated. At this point, her prednisone and mycophenolate mofetil were held. Infectious disease consultation was sought. Viral culture by unroofing a new vesicle on the right lower abdomen was taken and she was switched to intravenous acyclovir (250 mg every 8 h). Piperacillin-tazobactam was de-escalated to ampicillin-sulbactam (3000 mg daily), while continuing vancomycin. Lumbar puncture could not be done because of skin lesions over the site of the procedure.

Viral culture of the skin lesion came back positive for VZV, as well as a separate PCR test positive on the same lesion. The remaining infectious work-up included a negative repeat blood culture, serum cryptococcal antigen, negative SARS-CoV-2 PCR, negative urine histoplasma antigen, and negative HIV screen. Electroencephalogram study on day 4 revealed no epileptiform discharges.

From day 5 through day 11, despite gradual weaning off of sedation and pressor support, she continued to stay nonresponsive except for grimacing to painful stimulus. Further complications included worsening pancytopenia. Antibiotics were discontinued on day 10, while acyclovir was continued with the aim of at least 14 days. Goals of care discussion with family led to proceeding with comfort care, and the patient passed away.

## Discussion

BP is a common autoimmune subepidermal blistering disorder that has a broad clinical presentation. It typically presents as tense bullae and intense generalized pruritus [3]. BP patients have a high risk of systemic infections as many are elderly, receive immunosuppressants, or have functional impairments [4]. On the other hand, HZ is a viral infection that commonly presents as a painful vesicular rash. The skin lesions are less than 5 mm and are typically confined to one dermatome. DHZ is characterized by more than 20 lesions outside of a single dermatome. Delayed treatment, older age, and immunosuppression are all risk factors for the development of DHZ [1]. Diagnosis of disseminated shingles can be challenging as it can mimic other disseminated skin conditions and may result in treatment delay. Atypical manifestations include hemorrhagic, gangrenous, and less commonly bullous lesions [1], [5], [6], [7], [8].

A case control and cohort study suggested that patients with BP may have a 2.59 times higher risk for HZ reactivation relative to the general population as mentioned earlier, however, those patients were on corticosteroids or corticosteroids with tacrolimus [4]. Kamiya et al. reported a case of HZ who developed sudden onset tense blisters and edematous erythema for which BP titers were ordered given the suspicion of a BP presentation. Titers showed high anti-BP180 serum antibodies in association with the occurrence of HZ. They suggested that HZ reactivation may act as a triggering factor for a BP flare-up by enhancing BP180 antibodies [9]. The hypothesis behind this was a possible positive feedback loop between BP and HZ.

In our case, the patient was already on a maintenance dose of mycophenolate mofetil and had similar presenting BP flares in the past with unknown triggers while being on this medication. Her previous flares were treated with corticosteroids and showed a good response with complete resolution. Given the nature of her flares, it was presumed that this was merely another BP flare. The chronic maintenance dose of mycophenolate mofetil may have allowed VZV reactivation and dissemination. Initiation of the corticosteroids likely worsened the immunosuppressive state and accelerated the progression of disseminated zoster. The lesions were initially more localized to the L2-L3 dermatome of the right lower extremity and then became more widespread. The erythematous surrounding and base of the bullous lesions prompted us to initiate empiric antiviral therapy while investigating the lesions for shingles. The exact pathogenesis of bullae formation remains unclear; it may either be a distinct manifestation of VZV or due to the coinfection with exfoliative toxin–producing Staphylococcus species as some studies may have proposed [5], [6]. In our patient the history of BP adds another layer of complication in answering that question. It may suggest that the presence of bullae may be a reason to prompt us to investigate staphylococcal coinfection and should not be a reason to delay antiviral initiation [5].

A good primary prevention strategy that can be implemented is to offer vaccination for herpes zoster in all eligible patients with BP prior to administering high-dose immunosuppressive therapy. The current Centers for Disease Control and Prevention guidelines suggest two doses of the recombinant zoster vaccine (RZV, Shingrix) 2–6 months apart to prevent shingles and related complications in adults 50 years and older. Adults 19 years or older who are or will be immunosuppressed also need zoster vaccination, and the time between both doses may be shortened to 1–2 months [10]. Interestingly, our patient had received her live zoster vaccine 7 years prior to this presentation.

**Fig. 1:**
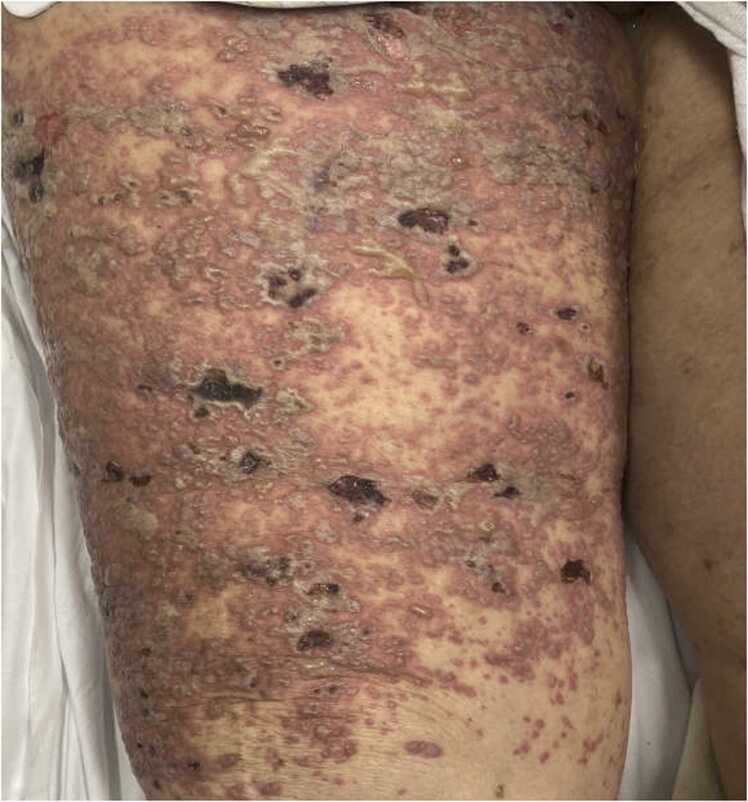
Showing erosive lesions circumferentially on the right thigh. Some lesions escharesque and crusted, while others were oozing yellow exudate.

**Fig. 2:**
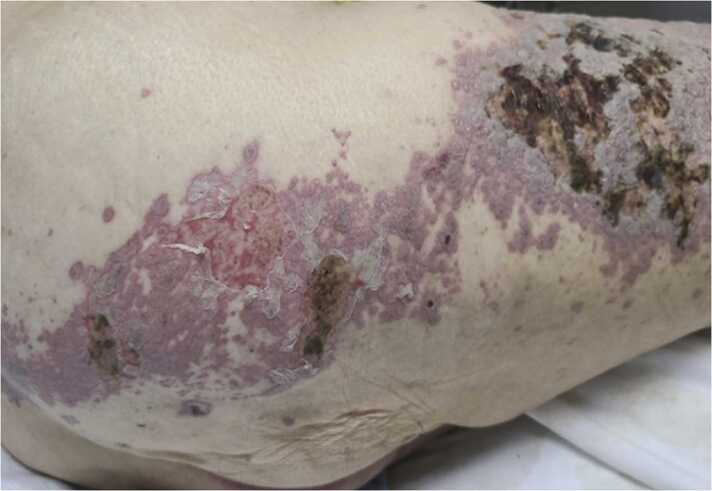
Lesions were widespread however predominantly concentrated over right thigh following a dermatomal distribution pattern.

While evaluating flare-up of autoimmune subepidermal blistering disorders, it is imperative to include viral reactivation syndromes as a potential triggering factor, especially since delay in timely initiation of antiviral therapy has a poor prognosis. Knowledge of atypical presentation of disseminated shingles and keeping a high suspicion in immunosuppressed patients is of paramount importance too [11]. Thus, maintaining vigilance in terms of DHZ detection and starting IV acyclovir early in the course may reduce the progression and mortality Fig. 1, Fig. 2.

## Funding

Not applicable.

## Ethical approval

No study performed onthe patient, did not require ethical approval.

## Consent

Obtained from the next of kin.

## CRediT authorship contribution statement

**Ahmed Shehadah**: Conceptualized the case idea, wrote, and edited the draft and manuscript, and collected data from the chart. **Alshaima Yousef:** Wrote the initial draft, and collected data from the chart. **Anas Hashem:** Edited the draft, reviewed the manuscript, and added references. **Amir A. Mahmoud:** Wrote the draft and manuscript, edited the manuscript, and added references. **Abhimanyu Aggarwal:** Reviewed the literature and edited the manuscript. All authors made substantial contributions to the final manuscript.

## Declaration of Competing Interest

The authors declare that they have no known competing financial or personal interests.
